# Metabolic Pathways and Targets in Chondrosarcoma

**DOI:** 10.3389/fonc.2021.772263

**Published:** 2021-12-06

**Authors:** Ida Micaily, Megan Roche, Mohammad Y. Ibrahim, Ubaldo Martinez-Outschoorn, Atrayee Basu Mallick

**Affiliations:** ^1^ Department of Medical Oncology, Thomas Jefferson University, Philadelphia, PA, United States; ^2^ Saint Francis Medical Center, Seton Hall University, Trenton, NJ, United States

**Keywords:** chondrosarcoma, metabolism, IDH, LdhA, lactate dehydrogenase, mTOR, PI3K - AKT pathway

## Abstract

Chondrosarcomas are the second most common primary bone malignancy. Chondrosarcomas are characterized by the production of cartilaginous matrix and are generally resistant to radiation and chemotherapy and the outcomes are overall poor. Hence, there is strong interest in determining mechanisms of cancer aggressiveness and therapeutic resistance in chondrosarcomas. There are metabolic alterations in chondrosarcoma that are linked to the epigenetic state and tumor microenvironment that drive treatment resistance. This review focuses on metabolic changes in chondrosarcoma, and the relationship between signaling *via* isocitrate dehydrogenase 1 and 2 (IDH1 and IDH2), hedgehog, PI3K-mTOR-AKT, and SRC, as well as histone acetylation and angiogenesis. Also, potential treatment strategies targeting metabolism will be discussed including potential synergy with immunotherapies.

## Introduction

Chondrosarcomas encompass a heterogeneous group of malignant cartilaginous matrix- producing tumors of the bone and are the second most common primary bone malignancy in humans, representing 25% of bone neoplasms (1). Histological grading and staging of chondrosarcomas are the most important factors to aid in prognostication. 85% of chondrosarcomas are histologically classified as conventional chondrosarcomas and can be subcategorized as central or peripherally located (1–3). The other 15% of chondrosarcoma histological subtypes include dedifferentiated, mesenchymal, clear cell and myxoid. Chondrosarcomas are graded from 1 to 3, or low, intermediate and high grade, based on cellularity, nuclear size, nuclear atypia, mitotic activity and matrix alterations (3). The majority of non-conventional chondrosarcomas include low and intermediate grade tumors that are slowly growing with low metastatic potential. However, 5-10% of chondrosarcomas are high-grade and this includes mesenchymal and dedifferentiated chondrosarcomas as well as a subset of conventional chondrosarcomas, which have a high metastatic potential and carry a poor prognosis (4).

The histological grade, stage, and tumor location determine treatment of chondrosarcomas. It is unclear if anticancer agents are active in chondrosarcomas emphasizing the need to understand drivers of cancer aggressiveness that may be the basis of novel therapeutics (2). Surgical management for localized disease is the mainstay of treatment but the specific surgical approach depends on the size, histological type and grade of chondrosarcoma. Small, atypical cartilaginous tumors/grade 1 chondrosarcoma are treated with intralesional curettage, and local high-grade chondrosarcoma are resected en-block (4). Conventional chondrosarcoma that is inoperable or widespread is routinely treated with chemotherapy due to its aggressive nature although it is unclear if any agents are active since there have been no high quality randomized controlled clinical trials in this disease. Chemotherapy was associated with improved overall survival for unresectable conventional chondrosarcoma in a retrospective study from the Rizzoli Institute in Bologna, Italy and Leiden University Medical Center in the Netherlands (3). Specifically, there was a survival advantage when being treated with chemotherapy at 3 years (OS of 26% with chemotherapy vs. 8% without chemotherapy, p<0.05) and the median overall survival was only 11 months for those patients that did not receive chemotherapy (3). However, it is unknown if selection bias contributed significantly to the differences in survival in this retrospective study. Another multi-institutional retrospective analysis with a 180 patients confirmed the poor outcomes for patients with unresectable chondrosarcoma with a median PFS of 4.7 months for first-line chemotherapy (4). The most commonly used chemotherapy regimens for chondrosarcoma are anthracycline-based regimens. However, there are no preferred regimens since there is a lack of positive randomized controlled trials and these tumors are generally chemo-resistant, with the exception of dedifferentiated and mesenchymal which may be partially chemo-sensitive (5).

Chondrosarcoma clinical research is focused on novel therapeutic approaches for inoperable, chemotherapy-refractory or metastatic chondrosarcomas. Although most cancer subtypes have multiple novel therapeutic options, chondrosarcoma lacks effective therapies with the exception of surgery and continues to be associated with poor clinical outcomes (4). Altered metabolism is a hallmark of cancers (6). A deeper understanding of chondrosarcoma metabolism, including glycolytic and TCA enzymes, metabolites, as well as signaling pathways, and tumor suppressor genes and oncogenes that modulate metabolism may identify vulnerabilities that could be exploited therapeutically (7). Genetic changes that affect mitochondrial metabolism are of particular interest in chondrosarcomas, since the most common mutations in these cancers are in the isocitrate dehydrogenase (IDH) gene, and the encoded mutant enzyme modulates mitochondrial metabolism (8). Specifically, mutations in IDH1 or IDH2 isoforms are identified in approximately 60% of chondrosarcomas (2). Also, glutaminolysis affects mitochondrial metabolism in a similar fashion to IDH mutations and is increased in chondrosarcoma (2, 6).

## Targeting Isocitrate Dehydrogenase (IDH) Mutations in Chondrosarcoma

IDH enzymes are the most commonly mutated genes in chondrosarcoma and the IDH1 isoform is more commonly mutated than IDH2 (8, 9). Mutant IDH-mediated epigenetic dysregulation leads to chondrocyte differentiation of mesenchymal stem cells and downregulation of osteogenic markers, which may explain the high and low prevalence of IDH mutations in chondrosarcoma and osteosarcoma respectively (10). Post-natal IDH1 R172Q mutations in chondrocytes in mice leads to enchondromas, which is a precursor lesion for chondrosarcoma (11). Knockout of both the mutated and wild type IDH1 *via* CRISPR–Cas9 from chondrosarcoma cell lines impaired anchorage-independent cell growth and migration with downregulation of integrins, which implicated mutant IDH1 in chondrosarcoma in the epithelial-to-mesenchymal transition with reduced growth rates *in vivo* and this phenotype was not rescued with restoration experiments with wild type IDH1 (12). Interestingly, mutations in IDH in high grade chondrosarcomas have been associated with CDKN2A/2B and TP53 alterations, prolonged relapse-free and metastasis-free survival (9). In contrast, a meta-analysis revealed that IDH mutations in chondrosarcoma are associated with poor outcomes (13).

IDH mutations are being targeted therapeutically in many cancer subtypes (8). IDH catalyzes the conversion of isocitrate to alpha-ketoglutarate (a-KG) and CO2 in the Krebs or TCA cycle (8). Three isoforms of IDH exist: IDH1 (cytoplasmic), IDH2 (mitochondrial-TCA cycle) and IDH3 (mitochondrial-TCA cycle). IDH1 and IDH2 are NADP-dependent enzymes that are mutated in 38-70% of conventional and dedifferentiated chondrosarcoma cases and induce a neomorphic enzymatic activity (6–13). The mutant forms of IDH1 and IDH2 require a-KG in order to produce a novel metabolite, D-2-hydroxyglutarate (D-2-HG), which becomes a competitive inhibitor in a-KG dependent enzymes and an oncometabolite, which activates hypoxia inducible factor (HIF) (11). HIF-1α expression is highly expressed in the majority of chondrsarcomas and is associated with poor outcomes (14). Also, HIF-2α is upregulated in human high-grade chondrosarcoma biopsies compared to low grade samples and gene amplification is associated with poor prognosis in chondrosarcoma patients (15). HIF-2α promotes chondrosarcoma tumor growth, invasion and metastasis using xenograft models *in vivo* and pharmacological inhibition of HIF-2α induces apoptosis (15). HIF-1α and HIF-2α are degraded in the proteosome *via* von-Hippel Lindau (VHL) and VHL disease is due to inactivating mutations in the VHL gene (pVHL), which leads to HIF-1α and HIF-2α stabilization (16). Chondrosarcomas have been described in patients with VHL disease (17–19). Also, low VHL expression is associated with higher grade, higher stage and worse outcomes in chondrosarcoma (20). In sum, IDH and HIF signaling is frequently altered in chondrosarcoma.

IDH1 and IDH2 mutated chondrosarcomas have a distinct metabolomic profile with increased lactate, the TCA intermediates succinate, fumarate, and malate, amino acids broadly and acylcarnitines compared to non-mutated cancer cells based on patient derived xenografts (21). This metabolomic profile of IDH mutated chondrosarcoma is consistent with increased glycolysis, anaplerotic flux into the TCA cycle and fatty acid oxidation (21) and is consistent with other studies that have revealed that there is HIF activation, high rates of glutaminolysis and lipid metabolism in chondrosarcomas (7, 15, 22, 23). Chondrosarcomas with high mRNA expression of glycolytic, TCA, glutamine catabolism and fatty acid oxidation genes were associated with poor outcomes in patients with chondrosarcoma (21).

Pharmacological inhibition of IDH1 reduced colony formation, migration, proliferation, and induced apoptosis in chondrosarcoma cells *in vitro* (24). However, a different group of investigators using other chondrosarcoma cell lines *in vitro* did not find that a mutant IDH1 inhibitor had an effect on proliferation and migration (25). IDH1 and 2 mutated chondrosarcoma cells have been shown to up-regulate glutaminolysis and glycolysis for the production of a-KG (25–27). High-grade chondrosarcomas have higher glutaminase expression although no significant differences were observed on the basis of IDH status (14). A subset of chondrosarcoma cell lines are sensitive to drugs targeting glutaminolysis and Bovee et al. studied the glutaminase inhibitor CB-839, the glutamate dehydrogenase inhibitor chloroquine as well as metformin and phenformin, which are inhibitors of mitochondrial oxidative phosphorylation (OXPHOS) and c-MYC and downstream of glutaminolysis (22). Interestingly, this study demonstrated that chloroquine increased apoptosis by increasing caspase 3/7 activity in three out of five cell lines (22). Metformin and phenformin reduced mTOR signaling in these chondrosarcoma cells by decreasing phosphorylated S6 (22) as has been previously demonstrated in other cancers (6). Metformin modulated autophagy with a decrease in LC3B-II levels. Metformin did not affect chondrosarcoma cell viability despite its known effects on mTOR signaling and mitochondrial respiration or OXPHOS. IDH2 mutations in chondrosarcoma cells are known to modulate mesenchymal differentiation through epigenetic dysregulation and treatment with the hypomethylating agent 5-azacytidine is a promising therapeutic strategy (15).

IDH1 inhibition in preclinical and clinical studies has had mixed results (12, 15, 16). A phase I multicenter clinical trial investigating the mutant IDH1 inhibitor ivosidenib (AG-120) in twenty-one patients with advanced chondrosarcoma demonstrated safety with minimal toxicity and a median progression free survival (PFS) of 5.6 months with a PFS rate of 39.5% at 6 months (28). 52% of the 21 patients had stable disease (28). There are ongoing phase I/II clinical trials of novel IDH inhibitors including AG-881 (NCT02481154) and AG-120 (NCT02073994). Aphase I/II clinical trial of metformin and chloroquine in IDH1/2-mutated solid tumors including three patients with chondrosarcoma (NCT02496741) did not find clinical activity (29). A clinical trial of the IDH-inhibitor AG-221 in chondrosarcoma is pending results (NCT02273739) (**Table 1**). In sum, mutant IDH causes metabolic reprogramming with production of D-2-HG that results in dysregulation of gene expression, differentiation status, DNA damage repair, inflammation, intracellular trafficking, ageing and cell death programs in cancer cells (8). Further research on mutant IDH inhibition is needed to determine its therapeutic value in chondrosarcoma.

**Table 1:** Clinical trials utilizing IDH and Hh pathway inhibitors in Chondrosarcoma.

AGENTS	STUDY DESIGN/PHASE	# of PATIENTS	PRIMARY EFFICACY ENDPOINT	REFERENCE
CB-839 (Glutaminase inhibitor)	Phase I	n=41	Safety/Efficacy	NCT02071862
Metformin/Chloroquine	Phase IB/II	n=38	Rate of disease control = 50%	NCT02496741 (29)
GDC-0449 (Hedgehog inhibitor)	Phase II	n=45	6 month clinical benefit rate	NCT01267955
IPI-926	Phase II	n=105	PFS, OS, ORR	NCT01310816
AG-221 (IDH2 inhibitor)	Phase I/II	n=21	Safety/Dose escalation	NCT02273739
AG-120 (IDH1 inhibitor	Phase I	n=170	Safety/Dose escalation	NCT02073994 (16)

## Targeting Lipid Metabolism in Chondrosarcoma

Lipids in cancer are thought to be protumorigenic due to their effects on epigenetic reprogramming, maintainance of redox balance by generating NADPH, reduction of ER stress/unfolded protein response, reduction of ferroptosis, effects as second messengers, pro-inflammatory effects, substrates for biomass production, and serving as catabolites to generate ATP (30). Also, increased amounts of cholesterol in the tumor microenvironment induce exhaustion in CD8+ T cells (31). Specifically, high levels of phospholipids and cholesterol are associated with aggressive cancer (32, 33). High cholesterol levels transform the lipid membrane and promote the activity of multidrug efflux pumps driving a multidrug resistant phenotype (33). Cholesterol acting as a second messenger activates mTOR and Hedgehog signaling (34). Conversely, mTOR activation induces denovo lipid and cholesterol synthesis (30, 35).

Inhibition of intracellular cholesterol biogenesis by deletion of sterol regulatory element–binding protein cleavage-activating protein (SCAP) reduces Hedgehog signaling and alters chondrocyte development (36). Statin drugs, which inhibit cholesterol synthesis, restore chondrocyte development and morphology (37, 38). There is upregulation of genes that are activated in cholesterol biosynthesis in IDH1 mutant chondrocytes (23). Consistent with increased biosynthesis, cholesterol levels are higher in IDH1 mutated chondrosarcomas (23). From a therapeutic standpoint, statins reduce cholesterol synthesis and administration of the statin lovastatin reduced chondrosarcoma progression *in vivo* with reduced proliferation and increased apoptosis rates (23). Hypoxia and starvation, as well as gene mutations in PI3K/AKT, RAS, and MYC have been implicated in upregulation of *de novo* lipogenesis, cholesterol synthesis and fatty acid desaturation (39). Autophagy is also related to lipid metabolism and there is upregulation in chondrosarcomas (40, 41).

The role of cholesterol and lipid homeostasis in chondrosarcoma is not elucidated. IDH1 mutations are known to cause metabolic reprogramming and regulate intracellular cholesterol biosynthesis. A mouse model utilizing IDH1 mutated chondrosarcoma cells identified an activation of cholesterol biosynthesis (21). IDH1/2 mutated chondrosarcoma cells treated with lovastatin reduced viability (42). These preclinical study suggests that targeting cholesterol metabolism may be promising in chondrosarcoma.

## Targeting Glucose Metabolism in Chondrosarcoma

Metabolism of chondrocytes using cell lines has been studied (17, 18). One of the earliest studies of metabolism of UDP-sugar metabolism in chondrocytes utilized Swarm rat chondrosarcoma chondrocytes to better understand the role of glycosylation in cartilage glycosaminoglycan and proteoglycan synthesis. This model demonstrated that stimulation through insulin or fetal calf-serum could increase biosynthetic activity of chondrocytes that can increase the demand of UDP-sugars in cartilage synthesis (43).

Glucose catabolism to C02 and water in the mitochondria occurs in the majority of cells, however under anaerobic conditions catabolism of glucose occurs exclusively in the cytosol and the end product of glycolysis is lactate. Aerobic glycolysis occurs at high rates in cancer cells and has been termed the Warburg Effect. Also, there is a greater appreciation of other cells that are glycolytic within the tumor microenvironment. The Reverse Warburg Effect describes when glycolysis in the cancer-associated stroma metabolically supports adjacent cancer cells. This catabolite transfer, which induces stromal-cancer metabolic coupling, allows cancer cells to generate ATP, increase proliferation, and reduce cell death (44).

Studies have been performed with inhibition of glycolysis in order to treat disease and overcome resistance to other anticancer agents. Multiple nodes in glucose metabolism and reactive oxygen species have been targeted in conjunction with conventional cytotoxic chemotherapy including doxorubicin, cisplatin and paclitaxel (19–23). Expression of lactate dehydrogenase- A (LDHA), which is a key glycolytic enzyme, in cancer cells has been associated with chemotherapy and radiotherapy resistance (2). Additionally, glycolysis can be suppressed by utilizing LDHA inhibitors such as oxamate and FX11 (2). Early studies focused on targeting glycolysis as a mechanism to overcome drug resistance were focused on LDHA. *In vitro*, paclitaxel resistance was overcome by utilizing the LDHA inhibitor oxamate which induced apoptosis (45). Given the high activity of LDHA and glycolysis in chondrosarcoma cells, LDHA may be a therapeutic target in chondrosarcoma. A cell line exposed to the LDHA inhibitor oxamate can overcome doxorubicin-resistance in chondrosarcoma (46). Another study demonstrated that the addition of oxamate to cisplatin helped overcome chemo-resistance in a chondrosarcoma mouse model (47). Also, the LDHA inhibitor, 1-(phenylseleno)-4-(trifluouromethyl) benzene (PSTMB) has been studied in models of colon cancer, lung cancer, breast cancer, hepatocellular carcinoma and has shown anticancer activity (48). Glucose metabolism may be a future therapeutic target in chondrosarcoma; although to our knowledge, no clinical trials are testing this therapeutic strategy.

## Targeting the Hedgehog Pathways in Metabolism of Chondrosarcoma

The hedgehog (Hh) protein regulatory pathway has been implicated in metabolic control, homeostasis and development (49, 50–52). The Hh pathway, which exerts potent metabolic effects, requires the appropriate control of Hh ligand production, processing, secretion and transport (49). A pathological role of the Hh pathway is associated with constitutive activation of transcriptional responses. Binding of Hh to the negative patched regulator (PTCH1) depressed positive smoothened regulator (SMO) in the cilia and phosphorylation and the cytoplasmic end, ultimately mediating downstream signal transduction (49). Glioma zinc finger transcription factors are activated by SMO, which translocate the transcription factors into the nucleus and bind to the promoters of several oncogenes. The disruption of this pathway drives tumorigenesis and glycolysis (49). Chondrosarcomas express high levels of hedgehog (Hh) proteins, and inhibition of the hedgehog pathway *in vitro* decreased proliferation of chondrosarcoma cells (49) and other solid tumors with down-regulation of P-glycoprotein (50). Additionally, the Indian Hedgehog (IHH)/parathyroid hormone related peptide pathway has been implicated in the pathogenesis of chondrosarcoma. IPI-926 (saridegib), a potent oral- Hh inhibitor, has been assessed in chondrosarcoma cell lines and has shown activity (51). Thereafter, another human chondrosarcoma cell line was also studied with Hh inhibition revealing decreased proliferation, invasion and migratory capacity (52, 53). IPI-926 was tested in a phase II study in chondrosarcoma that was stopped to accrual due to lack of efficacy (51). Another phase II, multicenter clinical trial was conducted on 45 patients with advanced, progressive chondrosarcoma utilizing the Hh pathway inhibitor GDC-0449 (vismodegib). The primary end point was a clinical benefit rate (CBR). The goal CBR was 40% at 6 months. However, the primary end point was not met, as the CBR at 6 months was 25.6% (95% CI 13-42%). Despite the negative results of this clinical trial, clinical benefit was observed in a subset of patients. A possible subpopulation of patients with grade 1 or 2 disease may benefit Hh pathway inhibition in combination with chemotherapy (**Table 1**). Previous studies have shown that the hedgehog pathway is dysregulated in chondrosarcoma and clinical trials evaluating Hh inhibition have shown that there could be a modest clinical benefit in chondrosarcoma (49–52).

## PI3K-AKT- mTOR Pathway in Chondrosarcoma

The mTOR pathway is a crucial regulator of cell metabolism, survival and proliferation (54). The growth factor regulated phosphoinositol 3-kinase (PI3K)-AKT signaling network has many direct and indirect downstream effects on cellular metabolism (6). Physiologically, PI3K-AKT pathway is activated in response to metabolic factors such as insulin, growth factors and cytokines to regulate glucose metabolism, biosynthesis of macromolecules and redox balance (54). These processes support metabolic homeostasis and the growth and metabolism of individual cells (55–58). Pathologically, oncogenic activation of PI3K-AKT reprograms cellular metabolism to anabolism and to promote cancer growth (59). AKT promotes glycolysis and glucose dependency for survival. Protein synthesis is also induced downstream of AKT through mTOR signaling (59).

The mTOR/PI3K pathway as well as IGFR-1 have been studied as a therapeutic anticancer target and this work has served as a foundation for studies in chondrosarcoma (55, 56). Inhibitors of the mTOR pathway have been utilized as single agents or in combination with other agents in both solid and hematologic malignancies (54–58). Human chondrosarcoma-derived cell lines demonstrated phosphorylation of receptor tyrosine kinases (RTKs), particularly AKT, MEK and S6 kinase suggesting clinical relevance of the PI3K/ATK/mTOR pathway in chondrosarcoma (54–58). RTK inhibition in the mTOR/PI3K pathway was studied preclinically utilizing a dual inhibitor, BEZ235 (54). Dual PI3K/mTOR inhibition was studied in a chondrosarcoma xenograft model and demonstrated significant suppression of growth utilizing BEZ235 and GCD-041 with rapamycin (54). Baicalin, a flavonoid extract utilized in traditional Chinese medicine, was found to induce apoptosis in a human chondosarcoma cell line *via* targeting of PI3K, AKT and mTOR pathways (57). Similarly, the mTOR pathway was studied in a preclinical rat chondrosarcoma model by utilizing the mTOR inhibitor everolimus which blocked cell proliferation as well as GLUT1 and HIF1a expression *in vivo (* 58). Activation of the HIF-1 pathway plays a central role in the adaptive response of tumor cells to hypoxia, and HIF-1 alpha expression is associated with high-grade chondrosarcomas and reduced disease free survival (60, 61). Everolimus reduced GLUT1 expression and HIF1a activity (60). However, single agent everolimus did not induce tumor regression in chondrosarcoma rat models (60). Additionally, doxorubicin was utilized in combination with everolimus and the combination had synergistic activity (61). One proposed mechanism for the synergy between everolimus and doxorubicin is by inhibiting both mTOR and AKT pathways simultaneously. The mTOR inhibitors sapanisertib and rapamycin have also been studied in chondrosarcoma cell lines and they induce decreased oxidative and glycolytic metabolism, independent of IDH status (7). Interestingly, chondrosarcoma cell lines treated with sapanisertib or rapamycin reduced cell viability with a decrease in glucose dependence and a shift towards glutaminolysis and fatty acid catabolism, and sapanisertib had the strongest response (57). Clinical studies of mTOR pathway inhibition in chondrosarcoma hold promise given the encouraging pre-clinical data.

## Targeting Angiogenesis in Chondrosarcoma

Tumors and require high rates of metabolite uptake from the vasculature to support their growth and have high vascularization (6). PI3K activation promotes vascular endothelial growth factor-A (VEGF-A) expression leading to angiogenesis, a key component of tumor growth and metastases (44, 62). Catabolites implicated in metabolic coupling include the monocarboxylates lactate, pyruvate, and ketone bodies. Monocarboxylate transporters (MCT) are critically necessary for release and uptake of these catabolites (63). MCT4 is involved in the release of monocarboxylates from cells, is regulated by catabolic transcription factors such as hypoxia inducible factor 1 alpha (HIF1A) and nuclear factor kappa-light-chain-enhancer of activated B cells (NF-κB), and is highly expressed in cancer cells as well as cancer-associated fibroblasts and has been shown to be highly expressed in sarcomas (63).

In chondrosarcoma human cell lines, angiogenesis is promoted by the fatty acid metabolic hormone adiponectin on VEGF-A (44). VEGF-A expression was induced by adiponectin through the utilization of the adiponectin receptor, PI3K, AKT, mTOR and HIF-1a pathways ^7,59.^ Pretreatment of chondrosarcoma cells with PI3K, AKT or mTOR inhibitors abolished the adiponectin effect on VEGF-A. As a previous study on chondrosarcoma cells demonstrated that adiponectin was linked to metastasis, this could be a possible treatment target in chondrosarcoma (62). The chemokine CCL5 has also been found to promote VEGF-A expression and angiogenesis in chondrosarcoma by activating the PI3K pathway (64). Inhibition of the PI3K pathway abolished the CCL5 effect on VEGF-A and angiogenesis (64). Targeting angiogenesis through multi-tyrosine kinase inhibitors was also studied in preclinical chondrosarcoma models (65). An inhibitor of VEFR2, PDGFR-B and FGFR1 through SU668 was used in a high-grade chondrosarcoma animal model (65).

Another mediator of angiogenesis, cyclooxygenase-2 (COX-2) and prostaglandin activity was studied in chondrosarcoma to determine if inhibiting its activity can improve chemotherapy and radiation sensitivity (42). Preclinical studies utilized agent celecoxib and NS-398 in four high-grade cell lines and although there was initial tumor regression, there was tumor relapse by 6 weeks (42).

A single-arm, multicenter phase 2 trial examining VEGF-inhibition by the oral tyrosine kinase inhibitor (TKI) pazopanib in metastatic or unresectable conventional chondrosarcoma was performed (66). Of the 47 enrolled patients, the primary endpoint of disease control rate (DCR) at 16 weeks was 43%, although this was not statistically significant (66). However, OS was 17.6 months and the median PFS was 7.9 months, which are promising time points in chondrosarcoma clinical trials. Grade 3 adverse events such as hypertension were observed, which are well-described with VEGF inhibitors (66).

As mentioned in **Table 1**, phase II studies investigating the safety and efficacy of multi-targeted tyrosine kinase inhibitors in various forms of chondrosarcomas are ongoing. These studies highlight the promise of targeting signaling pathways for the treatment of chondrosarcoma.

## Targeting the SRC Pathway in Chondrosarcoma

SRC proteins are cellular tyrosine kinases that have been studied in multiple solid tumor types and SRC modulates cell signaling, survival, angiogenesis, proliferation and migration. Cytoplasmic-RSC (c-SRC) contributes to receptor signaling from a number of growth factor receptors (47). Redox regulation of platelet derived growth factor receptor (PDGFR) stimulation, protein kinase C, PI3K and NADPH oxidase activity contribute to complex SRC kinase activation (67). In chondrosarcoma, negative regulators of the intrinsic or mitochondrial apoptotic pathway such as BCL2, which has crosstalk with SRC, are upregulated and mediate chemo-resistance in cancer (67). The multifunctional tyrosine kinase inhibitor Dasatinib (BMS-354825), which inhibits SRC and down-regulates BCL2 was studied in chondrosarcoma cell lines (66, 67). In chondrosarcoma, it has been studied in combination with doxorubicin in TP53 mutant chondrosarcoma cell lines (68). In the 8 doxorubicin resistant chondrosarcoma cell lines utilized in this study, SRC inhibition was found to overcome doxorubicin resistance by inducing apoptosis and inhibiting migration (68).

Another receptor tyrosine kinase target in chondrosarcoma is the platelet derived growth factor receptor (PDGFR), as preclinical data suggests that it’s signaling is induced in chondrosarcoma (67). Preclinical data has demonstrated that PDGFRa can mediate rapamycin-induced AKT phosphorylation in synovial sarcoma cell lines (67). As such, the PDGFR pathway was further studied in clinical trials utilizing single agent imatinib in chondrosarcoma without strong activity signals (see **Table 2**). The PDGFR pathway remains a promising therapeutic target in chondrosarcoma.

**Table 2:** Clinical trials utilizing Tyrosine Kinase Inhibitors (TKIs) in Chondrosarcoma.

TKI	STUDY DESIGN/PHASE	# OF PATIENTS	PRIMARY EFFICACY ENDPOINT	REFERENCE
Dasatinib	Phase II	n=366	RR, 6 month PFS	NCT00464620
Imatinib	Interventional	n=35	Tumor response	NCT00928525
Pazopanib	Phase II	n=70	ORR	NCT02066285
Regorafinib	Phase II	n=132	PFS	NCT02389244
Depsipeptide (Romidepsin)	Phase II	n=40	OR, time to progression, toxicity	NCT00112463

## Histone Deactylase Inhibitors in Chondrosarcoma

Histone dysregulation is a common future across multiple cancer subtypes. Histone acetylation helps alter the regulatory switch to disrupt homeostasis and cause tumor growth. Histone deacetylation and acetylation play key roles in chondrocyte differentiation. As such, histone deactylase inhibitors (HDAC inhibitors) have been well studied as treatment for various cancers, particularly chondrosarcoma (69). In a preclinical model, HDAC inhibitors were found to have antitumor effects by causing differentiation, apoptosis and inducing cell arrest (43). Subsequently, IDH wild type and mutant chondrosarcoma cell lines were studied with the combination of the HDAC inhibitor romidepsin and the glutaminase inhibitor CB-839 (22, 66). Another HDAC inhibitor, depsipeptide demonstrated antitumor activity in both chondrosarcoma cell lines as well as rat models (65). The promising results of these preclinical studies spurred further interest in clinical trials utilizing HDAC inhibitors, and we are awaiting results.

## Mitochondrial Metabolism in Chondrosarcoma

The mitochondria are the main source of ATP and the main site of generation of reactive oxygen species (ROS) and its effects have been studied in the progression of various malignancies. ROS is a second messenger notably used in tumor stemness, cell cycle progression, cell proliferation, cell survival, energy metabolism, cell motility, and angiogenesis (70). There is an interest in targeting ROS to induce cell death through induction of mitochondrial ROS production or alternatively using antioxidant strategies. Critical nodes in ROS production or antioxidant activity utilize cofactors such as NADPH, glutathione and FADH. 38-79% of chondrosarcomas have IDH1/IDH2 mutations with an enzymatic neomorphic activity and they use NADPH or NADH as a cofactor. The wild-type enzymes help with conversion of isocitrate dehydrogenase to α-ketoglutarate in cytoplasm and mitochondria respectively and when mutated they drive 2-hydroxyglutarate production, which is a metabolite that drives HIF-1α signaling (60). Hence, there is interest in evaluating the role of mitochondrial metabolism and the redox state in the treatment of chondrosarcoma (22).

The effects of targeting mitochondrial metabolism and apoptosis have been studied in human chondrosarcoma cell lines including studying effects of drugs on BCL2, BAX, Bid, cytochrome c and caspase-3 (71). The fungal metabolite Trichodermin from *N psidii* demonstrated anti-tumor activity by inducing mitochondrial apoptosis in chondrosarcoma cell lines (53). Other derivatives such as benzimadazole, benzofuran and honokiol have also been studied as pro-apoptotic agents (71–73).

Alterations in mitochondrial metabolism have also been implicated in resistance to chemotherapeutic effect. Translocase of the outer mitochondrial membrane complex subunit 20 (TOMM20) is an important subunit that is responsible for recognizing and translocating mitochondrial proteins into the mitochondria from the cytoplasm (62). Cells with greater mitochondrial mass and activity have greater TOMM20 expression. TOMM20 has been studied in human chondrosarcoma cell lines and found to have higher expression in higher grade chondrosarcomas, indicating a role of increased mitochondrial metabolism, glucose and lactose utilization and oxygen consumption (74). *In vivo*, TOMM20 induced larger tumor size. Additionally, TOMM20 overexpression was found to induce resistance to cell cycle specific chemotherapy agents (74).

## Immunotherapy in Chondrosarcoma

The altered metabolic landscape of tumor microenvironment can influence the suppression of immune cells and anti-tumor immunity (63). Metabolic dysregulation in cancer cells can also effect cell surface expression of proteins related to immune surveillance (75, 76). Specifically, PI3K-AKT-mTOR, HIF-1α and other pathways are implicated in immunotherapy resistance (63). The TCGCA does not include chondrosarcoma but IDH1 mutations have been associated with suppressed immune response pathways based on bioinformatics analyses of TCGA transcriptomic datasets (77). Chondrosarcomas are known to escape immune surveillance through the expression of immune checkpoints and express PD-L1/PD-L2 (78). Dedifferentiated chondrosarcomas are known to have tumor lymphocyte infiltration that correlates with PD-L1 expression and HLA expression, which suggest that these tumors might be sensitive to anticancer immunotherapy (78). Clinical trials utilizing immune checkpoint inhibition are listed in **Table 3**, and trials are ongoing to investigate the addition of immune checkpoint inhibition in conjunction with other immunotherapy or other novel agents. Preclinical studies of chondrosarcomas with hypomethylating agents in combination with immunotherapy with NY-ESO-1 and PRAME are promising (79). There is an ongoing phase I clinical trial utilizing this agent (see **Table 3**). Given the current ongoing research in sarcomas and immunotherapy (80), future directions for research may include incorporating metabolic agents in the treatment of chondrosarcoma with novel agents such as immunotherapy, T cell therapy and combination treatments.

**Table 3:** Immunotherapy in Chondrosarcoma.

AGENTS	STUDY DESIGN/PHASE	# of PATIENTS	PRIMARY EFFICACY ENDPOINT	REFERENCE
NY-ESO-1 specific T Cell Receptor (TCR) T Cell in Sarcoma	Phase I	n=20	Safety/Efficacy	NCT03462316
LN-145, aldesleukin	Phase II	n=80	To evaluate efficacy using ORR	NCT03449108
Pembrolizumab (anti PD-1)	Phase II	n=146	ORR	NCT02301039
Nivolumab plus Ipilimumab (anti PD-1 and anti CTLA4)	Phase II	n=40	CR and PR	NCT02982486
Toripalimab (anti PD-1)	Phase I	n=258	Safety/Efficacy	NCT0347640
Nivolumab plus ABI-009 (Nab-rapamycin)	Phase Ib	n=40	Safety, dose-escalation	NCT03190174

## Targeting Extrinsic Apoptosis in Chondrosarcoma

Apoptosis is the process of death and elimination of unwanted cells in the body as a normal part of growth and development. Cancer cells are known to evade apoptosis (76). Apoptosis can be mediated through the intrinsic pathway *via* BCL 2 or extrinsic pathway *via* pro-apoptotic receptors like death receptors (DR), such as DR4/DR5. INBRX-109 (a DR5 agonist) is one such pro-apoptotic receptor agonist drug that has been tested in chondrosarcoma with promising results (76, 81). Interim analysis of 12 patients with chondrosarcoma treated in the phase 1 expansion cohort with INBRX-109 showed a decrease in tumor burden by RECIST in 67% (8/12) of patients and disease control rate of 92% (11/12), and a phase 2 study is planned. We await these results to elucidate the role of targeting apoptosis in chondrosarcoma therapy.

## Conclusion

**Figure 1:**
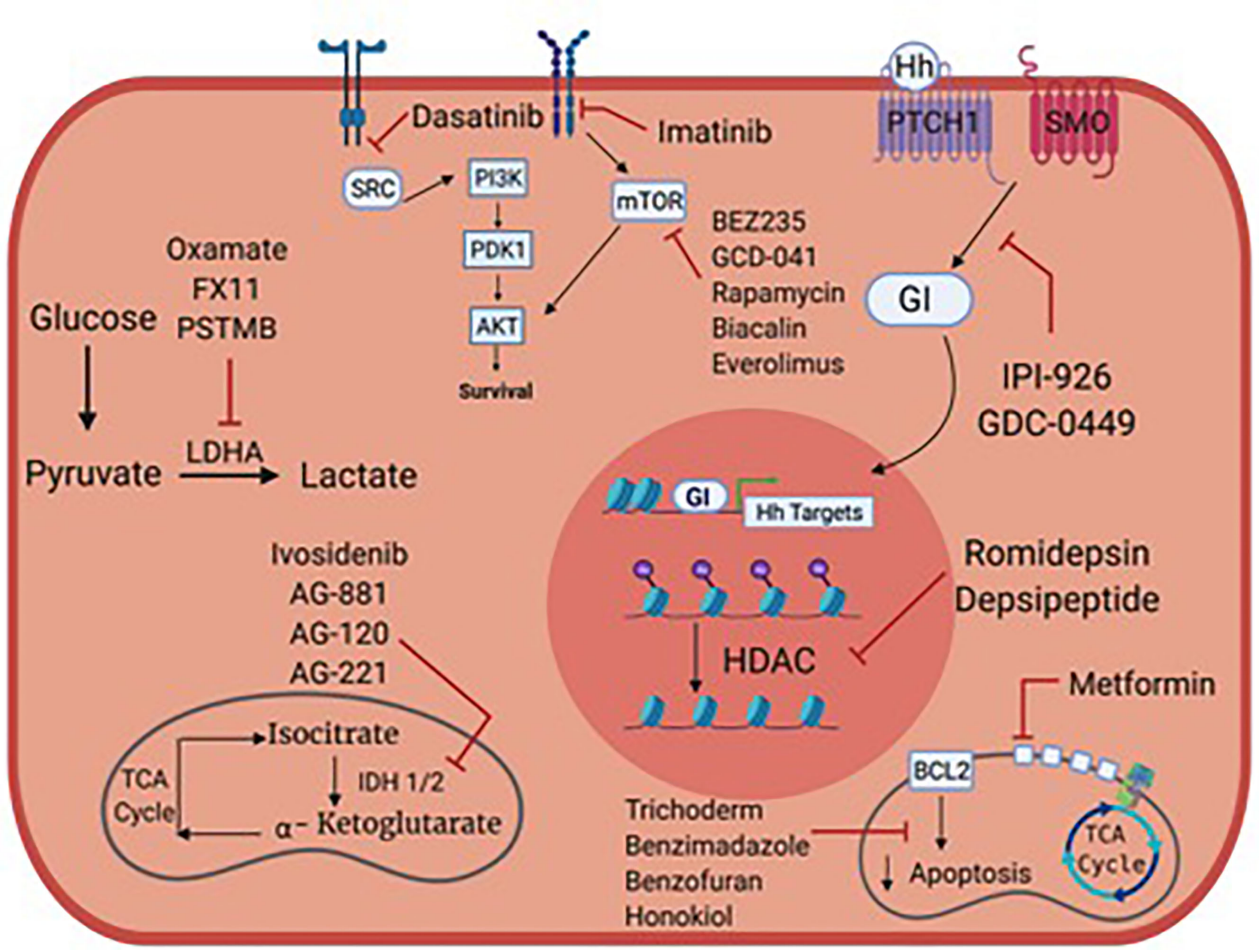
Metabolic targets and potential therapies in chondrosarcoma.

High grade, locally advanced and metastatic chondrosarcomas are frequently not amenable to surgery and are resistant to chemotherapy and radiation therapy. Preclinical studies focused on metabolism and/or oncogenic signaling pathways have led to clinical trials, but we still lack active anticancer agents in chondrosarcoma. **Figure 1** summarizes potential metabolic and therapeutic targets in chondrosarcoma. **Tables 1**–**3** list many relevant clinical therapeutic trials in chondrosarcoma. Targeting IDH mutations, lipid, glucose and mitochondrial metabolic targets, hedgehog, PI3K/Akt/MTOR, HIF and SRC pathways have yielded mixed results in clinical studies without clear therapeutic options in chondrosarcoma. While metabolic targets have been instrumental in understanding tumor development, the complex, heterogeneous roles of metabolism in chondrosarcoma likely renders mixed therapeutic results. As such, the therapy paradigm in chondrosarcoma aims to incorporate new approaches with anti-angiogenic agents and immunotherapy. Clinical trials will reveal the role of anti-angiogenic agents, immunotherapy and combination treatments and further research will be pivotal in illustrating their future therapeutic role with metabolic agents.

## Author Contributions

IM wrote, read and approved the final manuscript. MR edited and aided with images. MY aided in editing the manuscript and citations. UM-O wrote and edited the manuscript. AB wrote and edited the final manuscript. All authors contributed to the article and approved the submitted version.

## Conflict of Interest

The authors declare that the research was conducted in the absence of any commercial or financial relationships that could be construed as a potential conflict of interest.

## Publisher’s Note

All claims expressed in this article are solely those of the authors and do not necessarily represent those of their affiliated organizations, or those of the publisher, the editors and the reviewers. Any product that may be evaluated in this article, or claim that may be made by its manufacturer, is not guaranteed or endorsed by the publisher.

## Abbreviations

IDH, Isocitrate dehydrogenase; LDH, Lactate dehydrogenase; PD-1, Programmed-Death Receptor-1; PDL-1, Programmed-Death Receptor Ligand 1.
